# Operational scale entomological intervention for malaria control: strategies, achievements and challenges in Zambia

**DOI:** 10.1186/1475-2875-12-10

**Published:** 2013-01-08

**Authors:** Emmanuel Chanda, Victor M Mukonka, Mulakwa Kamuliwo, Michael B Macdonald, Ubydul Haque

**Affiliations:** 1 Department of Public Health and Research, National Malaria Control Centre, Ministry of Health, P.O. Box 32509, Lusaka, Zambia; 2 Department of Public Health. School of Medicine, Copperbelt University, P.O. Box 71191, Ndola, Zambia; 3 Global Malaria Programme, WHO Headquarters, World Health Organization, Avenue Appia 20, Geneva, 1211, Switzerland; 4 Department of Molecular Microbiology and Immunology, Johns Hopkins Bloomberg School of Public Health, Baltimore, MD, 21205, USA

**Keywords:** Malaria vector control, Integrated vector management, Policy and strategy, Community involvement, Private sector engagement, Monitoring and evaluation

## Abstract

**Background:**

While consensus on malaria vector control policy and strategy has stimulated unprecedented political-will, backed by international funding organizations and donors, vector control interventions are expansively being implemented based on assumptions with unequaled successes. This manuscript reports on the strategies, achievements and challenges of the past and contemporary malaria vector control efforts in Zambia.

**Case description:**

All available information and accessible archived documentary records on malaria vector control in Zambia were reviewed. Retrospective analysis of routine surveillance data from the Health Management Information System (HMIS), data from population-based household surveys and various operations research reports was conducted to assess the status in implementing policies and strategies.

**Discussion and evaluation:**

Empirical evidence is critical for informing policy decisions and tailoring interventions to local settings. Thus, the World Health Organization (WHO) encourages the adoption of the integrated vector management (IVM) strategy which is a rational decision making process for optimal use of available resources. One of the key features of IVM is capacity building at the operational level to plan, implement, monitor and evaluate vector control and its epidemiological and entomological impact. In Zambia, great progress has been made in implementing WHO-recommended vector control policies and strategies within the context of the IVM Global Strategic framework with strong adherence to its five key attributes.

**Conclusions:**

The country has solid, consistent and coordinated policies, strategies and guidelines for malaria vector control. The Zambian experience demonstrates the significance of a coordinated multi-pronged IVM approach effectively operationalized within the context of a national health system.

## Background

Malaria remains a leading cause of morbidity and mortality in sub-Saharan Africa [1]. Vector control interventions are expansively being implemented in endemic countries with unequaled levels of successes [2,3]. Effective and sustained vector control requires commitment from national autho-rities and funding partners [4]. Consensus on policy and strategy has stimulated unprecedented political-will, backed by international organizations and donors, culminating in setting of goals, indicators and targets, including ways of measuring progress towards their attainment [5-9].

Indoor residual spraying (IRS) and insecticide-treated bed nets (ITNs) remain the frontline interventions for malaria vector control [10-16]. In reducing abundance and infecti-vity of malaria vectors, these tools reduce overall transmission and protect individuals within a community [17,18]. The ownership and utilization of ITNs remain minimal [19] and the operational scale deployment of IRS is more complex than ITNs. There is mounting evidence that combining IRS and ITNs affords enhanced protection to exposed populations compared to using one method alone [14]. These core interventions can be supplemented in specific locations, by larval source management (LSM) strategies i.e. larviciding or environmental management [20-24].

Empirical evidence is critical for informing policy decisions and tailoring interventions to local settings. Due to anecdotal data, vector control tools are often based on assumptions i.e.: the rapid and significant impact of IRS for suppressing unstable malaria; the amenability of ITNs in effectively targeting the most vulnerable subgroups within communities with stable transmission; and, the greater operational and logistical ease of building and sustaining an ITN programme compared to an IRS one [25]. Thus, control programmes are encouraged to adopt the WHO-led integrated vector management (IVM) strategy [26], which is a rational decision making process for optimal use of available resources [27]. One of the key features of IVM is capacity building at the operational level to plan, implement and monitor and evaluate vector control and its epidemiological and entomological impact [28].

In Zambia, the major malaria vectors are *Anopheles gambiae*.*s*.*s*, *Anopheles arabiensis* and *Anopheles funestus*[29,30], with great heterogeneity in their transmission potential and spatiotemporal distribution [31,32]. Policies and strategies for malaria control are implemented according to recommendations set by the WHO with inherent monitoring and evaluation of malaria burden and trends, including tracking of the coverage and impact of interventions. This paper reports on the strategies, achievements and challenges of the past and contempo-rary malaria vector control efforts and provides guidelines for future deployment of entomological interventions in the country.

## Case description

The study was a retrospective analysis of routine surveillance data from the Health Management Information System (HMIS), data from population-based household surveys and various operations research reports.

### Operational design, status in policy and strategy implementation

The Zambian Ministry of Health (MoH) through the National Malaria Control Centre (NMCC) is responsible for the coordination and management of all vector control programs in the country. Transmission-reducing interventions (LLINs, IRS, Larviciding and Environmental management (EM)) are implemented and recorded at district level by the District Health Management Teams (DHMT) in collaboration with community health workers. All available information and accessible archived documentary records on malaria vector control in Zambia were reviewed to assess the status in implementing policies and strategies.

### Programmatic progress, epidemiological and entomological impact

A desk-based retrospective analysis was used to assess the progress in programmatic implementation, epidemiological and entomological impact of interventions. Programmatic progress and epidemiological impact was assessed through analysis of routine surveillance data from the HMIS, nationally representative cross-sectional population-based household surveys [32,33] and Demographic Health Survey (DHS) reports [34]. Data on malaria in Zambia is relatively complete with over 95% of districts regularly reporting quarterly to the HMIS until 2008. District-wise monthly malaria was reported from 2009 to present. Entomological research reports by multiple collaborating partners were reviewed to assess the impact of entomological interventions.

### Vector control policy and strategies

• Historical malaria vector control efforts. Malaria control in Zambia commenced in 1929 [35], and has progressed through several stages (Table 1). Pioneering interventions constituted environmental management and mosquito net use, coupled with diagnosis and treatment using quinine [22]. The success of vector control was enhanced by the enactment of statutory instrument “the Mosquito Extermination Act” [36,37]. Zambia first initiated IRS with DDT in the 1950s, at the same time malaria became a notifiable disease [38]. IRS coverage was reduced by 30% by 1973 and stopped in the mid 1980s [39]. With reduced vector control and the development of drug resistance [40,41] malaria cases increased from 121.5 per 1,000 in 1976 to 394 cases per 1,000 in 2002 [22].

• Malaria vector control policy change. In 1992, Zambia began health reforms and malaria control was prioritized in the basic health care package [38]. The NMCP developed its first National Malaria Strategic Plan (NMSP 2001–2005) with the vision “*reducing malaria morbidity and mortality by 50%”.* The policy emphasized prevention with ITNs [42]. In 2000, the private sector reintroduced IRS with pyrethroids and DDT [43] resulting in the NMCP to again implement IRS alongside LLINs [44]. Malaria was emphasized in both the fifth National Development Plan (NDP 2006–2010) and the National Health Strategic Plan (NHSP 2005–2009) [44]. In 2005, the NMCP developed a 2006–2010 NMSP with the vision *“A malaria-free Zambia*”, a theme of “*scaling-up for impact”* and the main goal of *“reducing malaria incidence by 75% and under-five mortality due to malaria by 20% by the year 2010”*. This policy included vector control using ITNs and IRS supplemented with LSM [45].

• Contemporary interventions. The current 2011–2015 NMSP seeks to provide the strategic framework for the NMCP, for the next five years. It has a vision of *“A malaria-free Zambia*” and a theme of *“consolidating malaria control gains, for higher impact”* and three main goals *“to reduce malaria incidence by 75% of the 2010 baseline, by 2015”, “to reduce malaria deaths to near zero and reduce all-cause child mortality by 20% of the 2010 baseline, by 2015”* and *“to establish and maintain five malaria-free areas in Zambia by 2015”*[46]. Vector control using ITNs and IRS supplemented with LSM remain key in this policy. The plan is linked to, and forms a part of the NHSP 2011–2015, and through it, to the Sixth NDP 2011-2015[46].

**Table 1:** Milestones in the history of malaria vector control in Zambia: 1929 to 2010


1929	Inception of malaria prevention and control efforts in Northern Rhodesia
1932	Malaria legislation initiated in Northern Rhodesia
1937	De Meillon research on vector behaviour (*An. gambiae *complex)
1944	Enactment of the Mosquito Extermination Act (environmental management)
1947	IRHS the Federal Malaria Eradication Programme in urban areas
1963	Split of Federation, Northern Rhodesia begins to lose resources to Southern Rhodesia
1964	Amendment of Mosquito Extermination Act (measures to reduce mosquito breeding)
1973	IRHS coverage in urban areas reduces by 30% and vector studies by Shelly conducted
1975	Chemoprophylaxis introduced in rural areas
1979	Studies on vector bionomics by Bransby Williams
1980	Mines reduce expenditure on malaria control
1985	UNICEF funded ITN project initiated in Samfya district
1992	Health reforms and inclusion of malaria in the basic health care package
1994	JICA funded ITN project in Chongwe district
1995	Annual in vivo surveillance commenced by NMCP, documentation of rising resistance to chloroquine, WHO funded ITN project in Ndola
1997	Signing of the WHO AIM Harare Declaration and implementation of the USAID and JICA funded integrated malaria initiative in three districts in Eastern province
1998	Extensive Malaria Knowledge, attitudes and practices (KAP) studies conducted across the country
1999	Malariometric surveys to define malaria endemicity and consolidation of the ITN distribution through the Community Based Malaria Prevention and Control programme in 41 districts
2000	Development of the first 2000–2005 National Malaria Strategic Plan, reintroduction of IRS by the private-sector and prioritization of ITNs for vector control by the malaria control programme
2001	Consultative discussions by the public sector with private sector and other stakeholders on IRS scale up
2002	Needs assessments for IRS implementation conducted in 5 districts and introduction of multiple ITN distribution mechanisms
2003	Treatment policy change from chroloquine to ACTs and reintroduction of IRS by the public sector
2004	Introduction of the IVM strategy, scaling up IRS to eight districts and the waiving of taxes and tariffs on ITNs and retreatment kits by the government
2005	Development of the 2006 – 2010 National Malaria Strategic Plan, strengthening of supervision, geo-coding and logistics for IRS by HSSP, SEA conducted in fifteen IRS districts and introduction of the free mass distribution of ITNs in Zambia. Environmental management for malaria control launched in Lusaka on 21^st^ October 2005
2006	Rapid scale up of ITNs for impact covering six of the nine provinces in the country and consultative meeting held with Valent Biosciences Coorporation (VBC) on larval source management using Bio-larvicides.
2007	Sockage pits, wash bays and evaporation tanks constructed in 15 IRS districts, efficacy studies on larvicides (*Bacillus thuringesis var.israelensis*, Insecticide Growth Regulators and Monomolecular Surface Films) conducted by the NMCC
2008	Public sector scales up IRS to thirty six districts, Production of guidelines on distribution and utilization of ITNs for Malaria Prevention and Control, Feasibility assessments for integrating LSM into the malaria control programme by Durham University, VBC and WHO conducted in Lusaka, Position statement on LSM made and Larviciding piloted in the urban areas of the initial five IRS districts, An inter-sectoral stakeholders consensus meeting on scaling up LSM to 8 urban districts held.
2009	Production of country specific guidelines for IRS in Zambia and scaling up the mass distribution of ITN to all the nine provinces, The use of larvivorus fish (*Gambusia affinis*) launched on 25^th^ April during the commemoration of the World Malaria Day, Needs assessments for scaled up LSM implementation conducted in eight urban districts using Global fund Round 4, insecticide resistance monitoring strengthened.
2010	IRS scaled up to fifty four districts, training and orientation of community and district health management teams on LSM and Implementationin May 2010, Monitoring and Supervision conducted in collaboration with Konkola Copper Mines and Mopani Copper Mines. Insecticide resistance management strategy for malaria vector control established

### Programmatic implementation

In Zambia vector control is implemented within the context of the IVM Global Strategic framework with strong adherence to its five key attributes [27,44]. Programmatic deployment of interventions follows clearly defined eligibility criteria based on local evidence in conformity with national guidelines [46]. IRS is predominantly targeted at urban and peri-urban areas. LLINs are targeted at rural areas. LSM is deployed during the dry season and confined to the urban and peri-urban areas where the breeding sites for malaria vectors are discreet and accessible [46].

**Figure 1:**
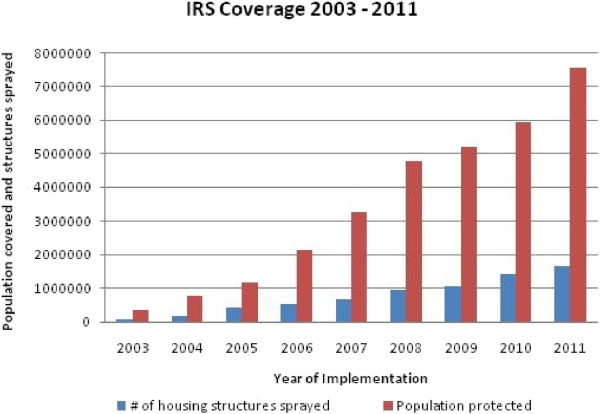
Progressive scale-up of indoor residual spraying from 2003–2011.

**Figure 2:**
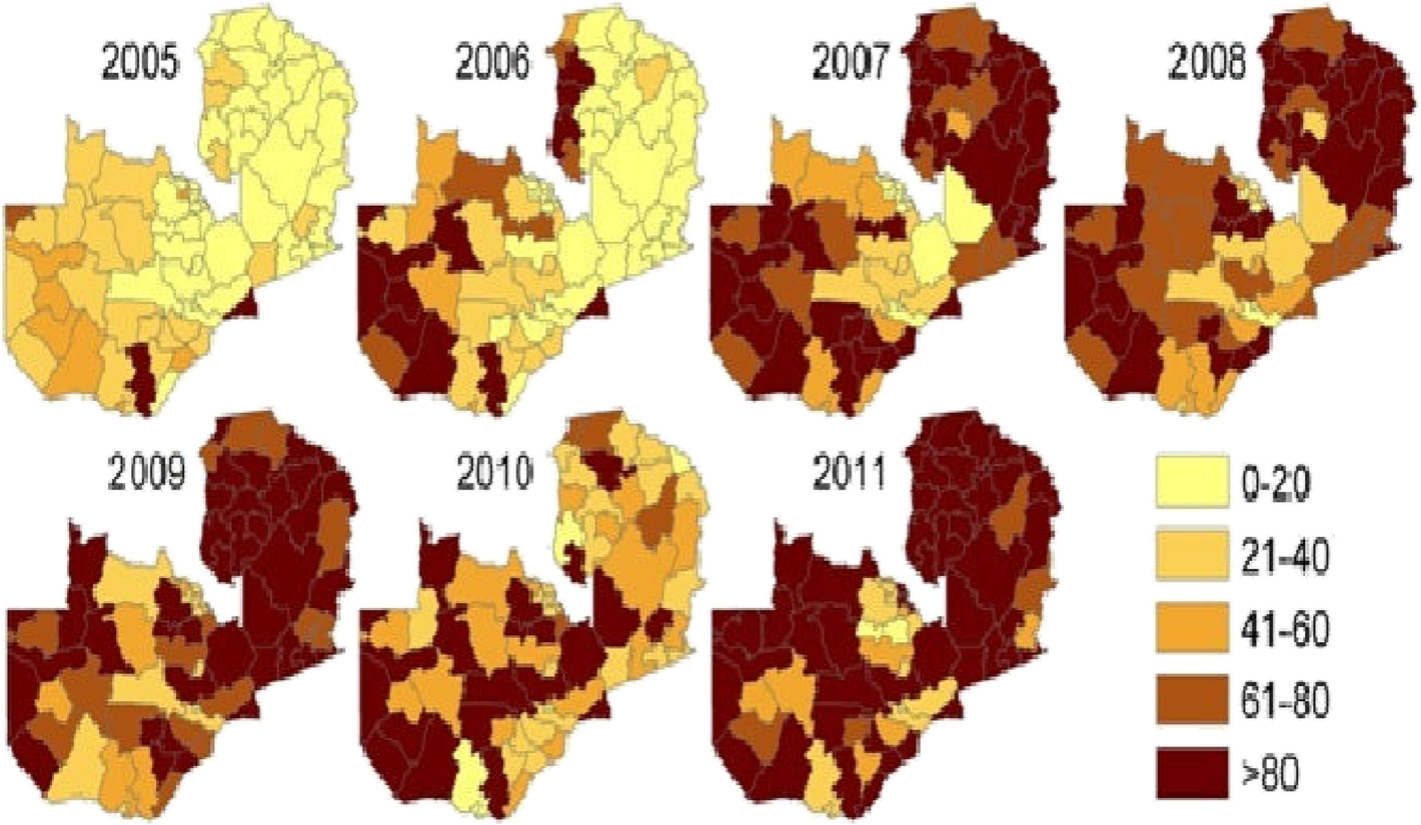
ITN distributions by district, representing percentage of district households receiving three ITNs per household (Source: 2012 National Malaria Control Action Plan).

• Indoor residual insecticide spraying. The operational design for IRS has an annual cycle [47]. The intervention has been expansively implemented with incremental scale up from 5 districts in 2003 to 36 in 2008 to 54 in 2010 and 72 in 2012 (Figure 1). With the goal of covering at least 85% of eligible households in targeted areas, IRS is deployed through annual campaigns using pyrethroids at 25 mg/m^2^ (deltamethrin and alpha-cypermethrin^,^ (Bayer); and lambda cyhalothrin, (Syngenta), carbamates (Bayer), organophosphates (Syngenta) and DDT at 2 g/m2 (Avima). The spraying is carried out prior to the peak malaria transmission that coincides with the rainy season from November to April [47]. Community Health workers (CHWs) and Neighbourhood Health Committees (NHCs) conduct the actual spraying operations under the supervision of DHMTs in targeted districts. Spray operations are in line with country specific guidelines, adapted from the WHO standard protocols [15,48]. Computerised spray management systems are used to continually monitor the progress and performance of spray operations [49].

• Insecticide-treated bed nets. The distribution of ITNs strives towards attaining a goal of universal (100%) coverage with at least 85% utilization rates in all eligible areas [46]. The coverage of ITNs has been increasing since 2000 via several distribution mechanisms, including ante-natal and child clinics, commercial, school health, equity, employer-based programmes and recently mass distributions of three nets per household since 2005 (Figure 2). Annual mass re-treatments campaigns were conducted during malaria commemorative day precedent to the national policy endorsement in 2007 to use and distribute only World Health Organization Pesticide Evaluation Scheme (WHOPES) recommended LLINs with at least 100 denier netting material [50]. More than nine million ITNs have been distributed with 75% of households possessing at least one net [51]. The distribution of ITNs is strictly in accordance with the country specific guidelines adapted from the WHO with a two component monitoring system (1) compilation of information on number of ITNs distributed and (2) tracking ITN coverage/ownership and utilization rates by households [50].

• Larval source management. Larviciding with *Bacillus thuringensis israelensis (Bti)* and *Bacillus sphericus (Bs)* and simple environmental modification and manipulation approaches i.e. canalisation, draining and land filling were reintroduced at programme level as supplementary tools to IRS and ITNs based on the stakeholders consensus meeting. This followed feasibility assessments for LSM conducted by Valent Biosciences Corporation, Durham university and the WHO [52]. The intervention was piloted in five districts and scaled up to eight urban districts [44], with further scaling up to 18 districts through collaboration partnership between Zambia and Cuban Governments i.e. microbial larvicides to reduce dependence on conventional insecticide based interventions. Implementation of the biolarvicides is through a multi-faceted approach with the full involvement of all organizations that impinge on vector proliferation with full participation of community based resource persons.

• Environmental and personal safeguards. Insecticide-based vector control requires sound environmental and personal safeguards and development of strong stewardship, institutional strengthening of in-country regulatory bodies is pivotal for protecting health and environment. Insecticides for IRS are regulated by the Environmental Protection and Pollution Control Act of Zambia that defines and mandates sound management of pesticides. Internationally, Zambia is party to the Stockholm convention on Persistent Organic Pollutants (POPs), which it ratified in 2006 and National Implementation Plans (NIPs) exist to ensure adherence to the treaty. The MoH ensures safe distribution and storage of vector control commodities and equipment through standardized and stringent stock management and control. All storage facilities need to conform to the WHO standard and should be licensed by the Zambia Environmental Management Agency (ZEMA), the in-country regulatory body. In the face of increased population [53], strengthened environmental and personal safeguards are necessary. A vector management plan has been developed as an annex to the Health Care Waste Management Plan.

• Monitoring and evaluation. In order to make evidence based decisions for vector control, systematic and improved monitoring and evaluation for both programmatic progress and outcome and impact is critical. In Zambia, deployment of tools has been streamlined through a geographical information system (GIS) based decision support [39]. Primary entomological indicators such as malaria vector species, densities, infectivity, resting and feeding behaviour, contact bioassays to determine the residual efficacy of insecticides and the quality of spraying and their insecticide resistance status are being monitored [39]. Epidemiological monitoring is conducted through routine surveillance reporting system and nationally representative cross-sectional population-based household surveys [33,34].

## Achievements

**Figure 3:**
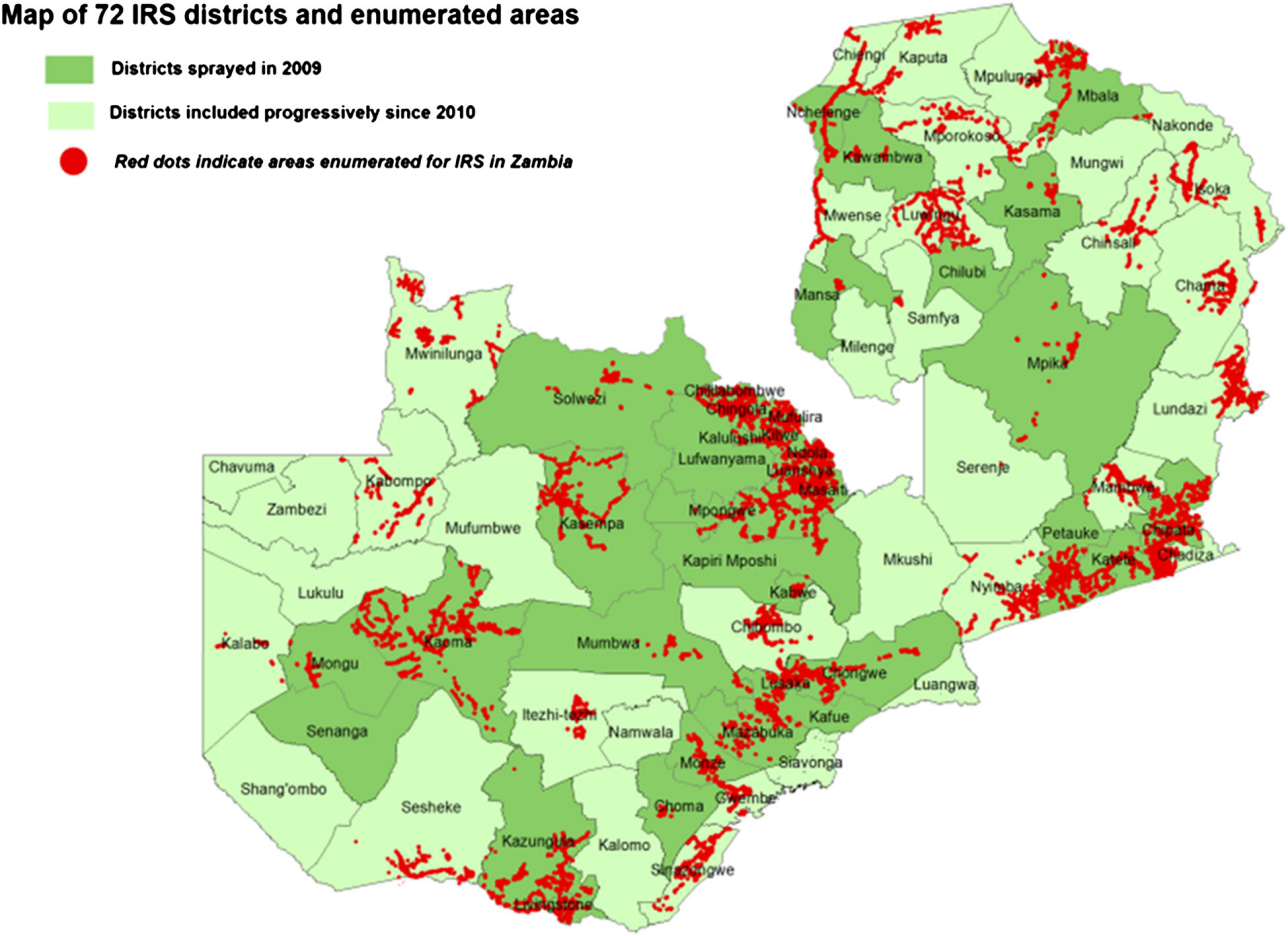
**Areas geo-coded for IRS implementation since 2009. **Map of 72 IRS districts and enumerated areas.

Programmatic progress is evidenced by remarkable increase in intervention coverage with over nine million LLINs distributed country-wide [33,34] (Figure 2). Household ITN ownership and utilization by children under the age of 5 years increased from 44% and 24% in 2006 to 68% and 41% in 2008 and to 73% and 50% respectively by 2010 (Table 1). The number of people covered by IRS has increased from 342,137 in 2003 to 5,951,303 in 2010 (Figure 1) with variation in percentage coverage of targeted households from 91.1% (95% CI = 90.9-91.3) and 90.1 (95% CI = 90.05-90.15) respectively, OR = 0.89 (95% CI = 0.87-0.91, *P* = 0.938) [54]. The GIS-based enumeration and geo-coding of structures (Figure 3) has rationalized the quantification of commodities and equipment for IRS [39]. Since 2010, DDT utilization for IRS has been discontinued in light of insecticide resistance development and adherence to the Stockholm convention on POPs. Epidemiological data indicate marked reduction in malaria related morbidity and mortality, a decrease in parasite prevalence among children under the age of five by 54.6% (95% CI = 34.7-73.1) and by 27.3% (95% CI = 13.2-48.2) from 2006 to 2008 and 2010 respectively, OR = 0.31 (95% CI = 0.089-1.100, *P* = 0.003) and a decline in the percentage of children with severe anaemia from 13.8% to 4.3% between 2006 and 2008 and 16% in 2010 (Table 2). In addition, national in-patient health facility data show that malaria cases and deaths were 55% and 60% lower respectively in 2008 when compared to the average in 2001 to 2002. Entomological impact is evident through a change in population structure of major malaria vectors, lack of sporozoite rates and thus loss of transmission potential [39].

**Table 2:** Programmatic progress with indoor residual spraying and insecticide treated nets (source MIS, 2006; 2008; 2010)

**Summary of progress with IRS and ITNs**	**MIS 2006**	**MIS 2008**	**MIS 2010**
Percentage of households receiving indoor residual spraying (IRS) in the previous 12 months among all households	9.5	14.9	23.1
Percentage of household members who slept under an ITN the previous night	19	34	42
Percentage of households covered by at least one ITN or recent IRS	43.2	68.3	72.9
Percentage of households covered by at least one ITN or recent IRS	43	68	73
Percentage of children ages 0–59 months who slept under an ITN the previous night	24	41	50
Percentage of children ages 0–59 months with malaria parasitaemia	22	10	16

## Challenges

In view of the scaling up, logistical challenges include inadequate transport and storage capacity at district level that invariably deter efficient delivery of both IRS and ITNs. Delays in disbursement of funds for IRS affects timely procurement and implementation of interventions, resulting in IRS not covering 100% of the earmarked households. Challenges with ITNs still remains low utilization (Table 2), lack of plans on disposal and replenishment of worn out nets, less efficacy and durability and abuse of nets e.g. in fishing areas. Continuous replacement of LLIN is not yet part of the national plan to maintain universal coverage of the intervention. There is no routinely collected national data regarding utilization of LLIN, this is only being captured in population-based surveys. With limited funding and skepticism surrounding LSM, its implementation remains a daunting task. The utilization of larval control in urban areas where the breeding sites are few, fixed and findable is still minimal. Currently, most vector control related IEC/BCC campaigns are in conjunction with malaria commemorative event such as World Malaria Day. The development of insecticide resistance in major malaria vectors in the country has great potential of compromising the efficacy of interventions [39]. While information regarding insecticide resistance is being collected and mapping conducted, the spatial scale of the collections remains small. There is limited investment in entomological related capital equipment and infrastructure such as storage facilities and laboratories. At provincial and district level, there is a lack of entomological capacity for optimal monitoring of interventions, funding to this component is not prioritized, with weak coordination and public-private sector involvement. Inadequate funding by the government invariably threatens sustainability of malaria vector control efforts. Generally, the malaria risk map has not been updated on a regular basis.

## Discussion and evaluation

Vector control has a proven record for saving lives by preventing, reducing or eliminating the transmission of vector-borne diseases [27]. As exemplified by the historical success of malaria vector control through the late 1970s in Zambia [20,22] which was not devoid of inequities. While the amendment of the mosquito extermination act in 1964 contributed markedly to compliance of the public members [38], intervention effects on the malaria burden where more significant in urban areas than among the rural populace. In order to rapidly scale-up country-wide, the Zambian NMCP has refocused its strategic approach towards ensuring that goals and objectives of increased access and utilization of proven interventions are met [46]. There is great variability in policies and strategies between historical and contemporary malaria control efforts in the country (Table 3).

**Table 3:** Comparisons in strategies, achievements and challenges between historical and contemporary vector control efforts

**Attribute**	**Historical**	**Contemporary**
Strategies	LSM (simple EM and Larviciding) as main thrust interventions and IRS as supplementary.	Primarily based on IRS and ITNs as frontline tools, supplemented with LSM
	Targeted vector control interventions confined to urban areas with limited political will.	Nationwide universal coverage with vector control tools deployed in both rural and urban areas with enhanced political commitment.
	Implementation by full time public health workers from the Mines, MoH and Local authorities.	Evidence based implementation by community based resource persons.
Achievements	High coverage of interventions reducing malaria disease to a notifiable level in operational settings	Strong inter-sectoral collaboration between private and public sector with appreciable impact on the disease in both rural and urban areas
	Monitored species composition, their relative densities and sporozoite rates in vector populations	Robust entomological monitoring of impact with a rational insecticide resistance management strategy
	Full commitment by municipalities with enhanced with the enforcement of strong statutory instruments	Strong monitoring & evaluation and IEC. Geo-coding of structures, efforts to determine the impact of interventions on vectors
Challenges	Confined to urban areas, lack of advocacy and social mobilization.	Lack of utilization of ITNs and abuse. Lack advocacy on supplementary interventions resulting in limitation in their funding.
	Weak and limited environmental and personal safeguards	Increased population, coordination at provincial and district levels
	Limited entomological monitoring including impact of interventions on vectors	Limited enforcement of statutory instruments, lack of total commitment by municipalities.

The deployment of an effective and evidence-based malaria vector control requires locally informed decisions as the epidemiology of the disease varies at a small scale, suggesting the need for precise targeting [26]. To this effect the programme has made great progress in implementing WHO-recommended policies and strategies, and taking into account the interventions that are appro-priate in different epidemiological settings. Cognizant of the heterogeneities in operational settings, deployment of scientifically proven high impact vector control tools is within the context of the Global Strategic framework for the IVM strategy with strong adherence to its five key attributes: *Advocacy, social mobilization and legislation; Colla-boration within the health sector and with other sectors; Integrated approach; Evidence-based decision-making* and *Capacity-building*[27,44]. Zambia has solid, consistent and coordinated policies and strategies for malaria vector control in place with engagement of communities in deploying tools [46]. In response to the international calls to protect both human health and the environment from DDT through the United Nation Environment Programme (UNEP), Global Environment Facility (GEF) and WHO, together with insecticide resistance development, Zambia has halted the use of DDT for IRS.

Programmatic scaling-up of WHO-recommended vector control tools have been intensified with substantial scores, as compared with set international targets. However, vector control is not a sole preserve of the ministry of health alone but requires involvement of various stakeholders including community engagement [27]. The IVM strategy has enhanced inter-sectoral collaboration and strengthened public-private sector partnership [44]. There is unprecedented political will and huge partnership support operating within the principle of three ones: one coordinating mechanism; one implementation plan and one monitoring plan. Strengthened collaboration has leveraged resources from World Bank, MACEPA, GFATM, JICA, IVCC and USAID/PMI. Geo-coding of structures and of breeding sites earmarked for interventions has streamlined quantification of commodities and equipment and has resulted in timely procurements as well as implementation. The Government policy to waive all taxes and tariffs on ITNs has reduced the price of ITNs in the commercial sector and administrative costs for routine ante-natal care and child clinic distribution [47]. The distribution of nets from ports of entry directly to the earmarked districts is striking. These achievements can be attributed to increased advocacy, communication and behaviour change, efficient partnership coordination including strong community engagement, increased financial resources and evidence-based deployment of key technical interventions in accordance with the national malaria control programme policy and strategic direction [46].

In the wake of scaled up transmission-interrupting strategies, monitoring and evaluation of the vector control interventions is an indispensable underpinning for rational and evidence based IVM approach. The historical vector control efforts where characterized with limited entomological monitoring and lack of comprehensive studies on impact of interventions on malaria vectors. Currently, there is strong evidence-based monitoring and evaluation to facilitate for the documentation of progress made towards the achievement of international goals and targets by 2015 (Table 3). Over the last 10 years in Zambia, strengthened operational research through the GIS-based decision support system (DSS) has improved the routine tracking of entomological and parasitological indicators and provided the epidemiological impact of ITNs and IRS [39]. However, limited entomological capacity for surveillance to date has restricted the detection of potential temporal changes in vector bionomics throughout the country.

While there is enough capacity for planning and deploying vector control tools at provincial and district level in Zambia, there is need to further strengthen capacity for entomological and epidemiological monitoring of interventions. Particularly, establishment of entomological capital equipment and infrastructure capacity at these levels, including increased human resource training to be able to drive forward the malaria vector control agenda. This will not only maintain the required capacity for implementation, given the high levels of human resource attrition, but facilitate for the tracking of primary entomological parameters which are critical for guiding deployment and impact assessment of interventions.

Maintaining the momentum and the gains is critical as the programme strives to achieve universal coverage of evidence-based and proven interventions. With the development of insecticide resistance and the potential shift in malaria vector bionomics i.e. indoor biting to out door biting, the current vector control methods are not devoid of limitations. To sustain the efficacy of the vector control tools, commitment towards conservation of the limited arsenal of insecticides by their judicious use through a rational insecticide resistance management strategy guided by regular and expansive insecticide resistance surveillance and mapping together with increased monitoring of malaria vector bionomics is critical [55]. There is need to estimate malaria transmission intensity to compare and interpret malaria interventions conducted in different places and times and to objectively evaluate options for malaria control [56]. The entomological impacts expected from any entomological intervention are vectorial capacity, entomological inoculation rates and the basic reproductive number [57,58]. Attainment and maintenance of set goals requires continuous surveillance, monitoring and evaluation to make informed decisions and guide control efforts.

In light of the challenges of the dwindling financial resources that have followed in the wake of diminishing donor support and the limited Government funding for malaria control, streamlined uptake and purposeful deployment of key vector control tools requires efficient utilization of supportive strategies. To further improve coverage and utilization rates of interventions; a regularly updated, interactive, comprehensive and sustained national advocacy, IEC/BCC campaign, and a viable operations research feeding into and providing timely and sound evidence to guide implementation and inform policy decision-making are critical. To ensure and sustain adherence to high standards of supervision monitoring and evaluation, and adherence to personal and environmental safeguards, legal standards and guidelines is cardinal.

The IVM strategy requires reconsidering the combination of vector control methods over time, as the environment, epidemiology and resources change [59]. Even with intensive vector control, there is still some heterogeneity in the levels of malaria endemicity in Zambia. To further reduce the disease burden in both high and low transmission settings in the country, there is need to explore synergies and to ensure integration of vector control activities [44]. This would require institution of outdoor interventions like LSM. As evidence continues to accumulate suggesting that some urban localities are becoming low-transmission areas, integration of LSM strategies to complement IRS and ITNs, may assist to clear the residual transmission in a cost effective manner. In addition to enhanced operations research, synchronizing deployment of effective and sustainable entomological tools in the context of cross boarder initiatives is also essential. While sustaining strong national IEC/BCC campaigns through interpersonal and community-based approaches to increase the demand for the correct and consistent use of LLINs is critical, further research into novel vector control approaches should be encouraged [55].

## Conclusions

In response to the increasing burden of malaria and the call by the WHO for scaled up implementation of proven vector control interventions, coupled with the unprecedented availability of resources for vector control, the Zambian NMCP has made progress in setting up strategies, scaling up programmatic implementation of interventions and monitoring their impact on malaria control. Sustained delivery of effective and evidence based vector control interventions has been informed by emerging evidence from ongoing operational research coupled with strengthened advocacy, social mobilization and political leadership. To attain the goal of elimination increased inter-sectoral collaboration and community involvement, strengthened supervision, technical and operational coordination and collaboration including cross boarder initiatives and resources mobilization is cardinal to reduce transmission. The Zambian experience demonstrates the significance of a coordinated multi-pronged IVM approach effectively operationalized within the context of a national health system.

## Abbreviations

ACT: Artemisinin-based combination therapy; BCC: Behaviour Change Communication; CHW: Community Health workers; DDT: Dichloro Diphenyl Trichloroethane; DHMT: District Health Management Team; DHS: Demographic Health Survey; DSS: Decision Support System; EM: Environmental Management; GEF: Global Environment Facility; GFAT: Global Fund to fight AIDS/Tuberculosis and Malaria; GIS: Geographical Information System; HMM: Home Management of Malaria; HMIS: Health Management Information System; HSSP: Health Systems Strengthening Programme; IEC: Information Education and Communication; IRS: Indoor Residual Spraying; IVCC: Innovative Vector Control Consortium; IVM: Integrated Vector Management; JICA: Japanese International Corporation Agency; LLINs: Long-Lasting Insecticidal Nets; LSM: Larval Source Management; MACEPA: Malaria Control Evaluation Programme in Africa; MIS: Malaria Indicator Survey; NDP: National Development Plan; NHSP: National Health Strategic Plan; NHC: Neighbourhood Health Committees; NIP: National Implementation Plans; NMSP: National Malaria Strategic Plan; NMCC: National Malaria Control Centre; NMCP: National Malaria Control Programme; PMI: Presidents Malaria Initiative; POP: Persistent Organic Pollutants; RBM: Roll Back Malaria; RDT: Rapid Diagnostic Tests; SEA: Supplementary Environmental Assessment; UNEP: United Nation Environment Programme; USAID: United States Agency for International Development; WHO: World Health Organization; WHOPES: World Health Organization Pesticide Evaluation Scheme; ZEMA: Zambia Environmental Management Agency.

## Competing interests

The authors declare that they have no competing interests.

## Authors’ contributions

EC: Conceived the idea, collected and analysed the data, and drafted the manuscript. VMM, MK and MBM: Critically reviewed the manuscript. UH: Guided data analysis and interpretation and contributed to the drafting of the manuscript. All authors read and approved the final manuscript.
